# Introducing a Mobile-Connected Umbilical Doppler Device (UmbiFlow^™^) into a Primary Care Maternity Setting: Does This Reduce Unnecessary Referrals to Specialised Care? Results of a Pilot Study in Kraaifontein, South Africa

**DOI:** 10.1371/journal.pone.0142743

**Published:** 2015-11-23

**Authors:** Josef Mufenda, Stefan Gebhardt, Rita van Rooyen, Gerhard Theron

**Affiliations:** 1 Department of Obstetrics and Gynaecology, Stellenbosch University and Tygerberg Hospital, Cape Town, South Africa; 2 mHealth Inc., 12525 El Camino Real B, San Diego, California, 92130, United States of America; INRA, FRANCE

## Abstract

**Objectives:**

UmbiFlow**™** is a mobile-connected Doppler device that utilises a continuous waveform to measure resistance in the umbilical artery. The main aim of this pilot study was to determine whether the use of UmbiFlow™ for umbilical artery Doppler in patients with a suspected decreased symphysis fundal (SF) growth could safely lead to a decreased number of patients requiring referral to a more specialised level of care. A secondary aim of the study was to evaluate the effectiveness of UmbiFlow™ Doppler as a screening tool for concealed placental insufficiency in late bookers by using a single screening cut-off value that will be abnormal for any gestation >28 weeks.

**Methods:**

The cohort comprised two groups of patients: The first group included all follow-up patients with suspected intra-uterine growth restriction (a decreased symphysis-fundus measurement based on serial assessment) who underwent on-site UmbiFlow™Doppler testing performed by the midwife directly after the clinical examination. The second group included late bookers, where gestation was uncertain; but estimated >28 weeks based on clinical grounds. This group was comprised of unselected patients who report to antenatal care late for the first time and received an UmbiFlow™Doppler test for concealed placental insufficiency.

**Results:**

UmbiFlow™Doppler could reduce the number of false referrals to hospital by 55%. A single UmbiFlow™Doppler test in late bookers appeared to identify a group of women at moderate risk of lower birth weight babies.

## Introduction

Fetal growth restriction is defined as an intra-uterine fetus with an estimated fetal weight <10^th^ percentile for gestational age [1]. Into these 10% of the pregnant population, the challenge is to identify the babies that have pathological growth deficiency [2]. There is still controversy regarding the appropriate diagnosis and management of intra-uterine growth restriction (IUGR), especially for early-onset growth restriction [3]. There are many causes for fetal growth restriction, but the most important one is sub-optimal placental perfusion due to abnormal placentation (placental insufficiency) [1]. Histological examination of the placentas of babies with growth restriction and abnormal uterine artery Doppler show significantly more intervillous thrombi and villous infarctions than those with normal Dopplers [4].

Fetal growth in the Tygerberg Hospital catchment area is monitored by centile charts customised for the inhabitants of the Western Cape [5] [6]. In a low-resource setting, without access to modern ultrasound evaluation, the use of serial measurements of the symphysis-fundus (SF) distance is a reliable surveillance tool to detect babies that are small for their gestational age. Women are referred if a single SF measurement is below the 10^th^ centile for gestation, or when serial measurements cross centiles. The strength of SF measurements lies in serial assessment, but that does introduce inter-observer variation and the overall evidence on fundal height measurement as a predictor for IUGR is still mixed [7].

The major causes for perinatal loss at Tygerberg Hospital are abruptio placentae (29% of deaths), fetal anomalies (13%), hypertension (10.7%), preterm labour (9%) and infections (9%); and idiopathic intra-uterine growth restriction (6.3%) [8]. In this specific hospital population, 41% of babies that die from abruptio placentae are also small for gestational age [9]. It is thus extremely important to try and identify those babies at risk of ischaemic placental disease, the syndrome that links preeclampsia, intrauterine growth restriction, and placental abruption [10]. However, in a low risk pregnant population without prior history of stillbirth, growth restriction or hypertension, the most reliable way of screening for ischaemic placental disease is with fundal growth. Poor fundal growth as measured by serial SF growth are detected in 5.4% of low risk pregnancies in this population and Doppler tests of the umbilical artery (UA) in these patients are reliable to detect those babies at risk for placental insufficiency [11]. More importantly, the specificity of Doppler in this setup is high (97%); making it an ideal surveillance test in a low resource setting [12].

However, it is widely accepted that UA Doppler is much more reliable (higher sensitivity) in a high-risk, selected population. A meta-analysis of randomised trials showed no reduction in perinatal mortality when routine UA Doppler was added to the management of low-risk women [13]. The Cochrane review of a low-risk population with normal SF growth (in serial assessment) showed no advantage for the additional use of UA Doppler, although the number of participants in this meta-analysis is small and more information is needed to make a definite conclusion on the absence of effect [14]. In recent years, late-onset intra-uterine growth restriction (IUGR) has appeared as an important cause for perinatal mortality and morbidity and researchers had intensified the search for a reliable test to detect IUGR in otherwise low-risk women [15]. Bolz et al, in a study on UA Doppler in low-risk pregnancies indicated that a raised umbilical artery resistance was significantly associated with small for gestational age babies as well as increased Caesarean section rates [16]. An UA resistance index (RI) of >0.5 between 37 and 41 weeks in this population correlated with an increase in Caesarean section rate.

The predictive value of UA Doppler in suspected small-for-gestational-age babies in late pregnancy is still unclear. However, it is re-assuring that in growth-restricted fetuses with normal flow in the UA, the incidence of adverse perinatal outcome is relatively rare and usually followed a pathology unrelated to the growth restriction [17]. In a study by Fillmar et al, measuring UA Doppler at 36 weeks in an unselected hospital population, there was an increase in adverse outcome with abnormal UA Doppler, even in appropriately grown fetuses [18].

When gestational age is uncertain, it becomes less accurate to determine poor SF growth in later pregnancy. Although a normal UA Doppler at this gestation may not exclude growth impairment [19], an abnormal value at this gestation may be valuable as a referral tool for more intensified growth assessments or other tests for fetal wellbeing.

Most high-end ultrasound machines feature a Doppler mode and thereby have the ability to measure the RI in the UA, using pulsed wave Doppler. Continuous wave systems use continuous transmission and reception of ultrasound and can measure high blood flow velocities, but cannot be paired with a simultaneous ultrasound image as in pulsed wave modalities. Continuous wave machines are also much cheaper. The Medical Research Council (MRC) Unit for Perinatal Mortality, the MRC and the Centre for Scientific and Industrial Research (CSIR) have developed an innovative and affordable continuous-wave mobile-connected Doppler analyser dubbed UmbiFlow™ [20]. It consists of a handheld proprietary vascular transducer (probe) with a universal serial bus (USB) cable that connects to any Windows-based computer on which the necessary software is installed. It outputs a 4 megahertz ultrasonic beam at a hardware limited ultra-sonic power output of 20 milliwatt. An integrated 3G (third generation mobile telecommunications technology) card facilitates a mobile internet connection and automatic upload of examination results to a central server for remote expert support and electronic health record management. The system is inexpensive and users can be trained in its usage within a few days.

**Fig 1:**
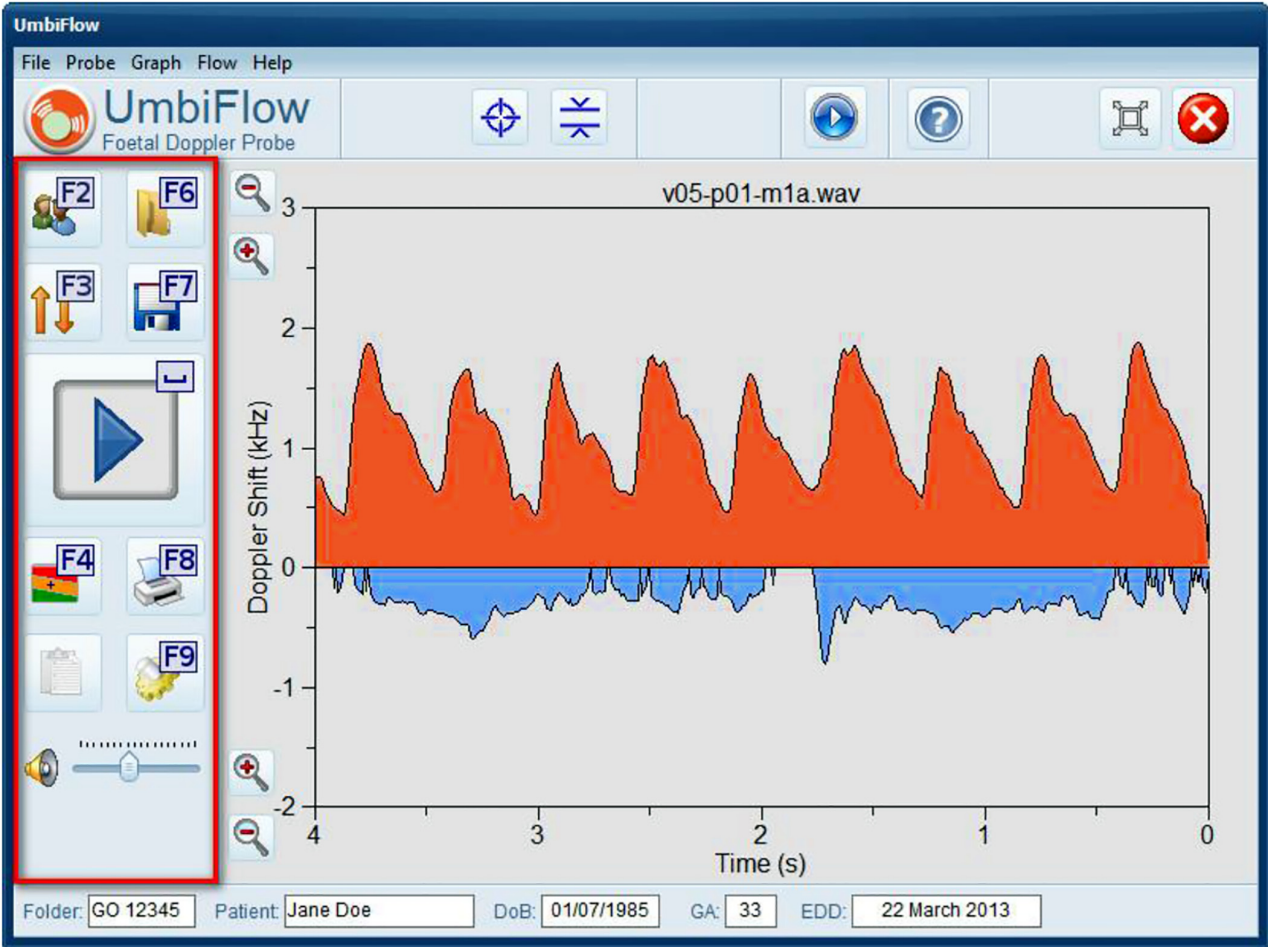
User interface of UmbiFlow™ Doppler software.

**Fig 2:**
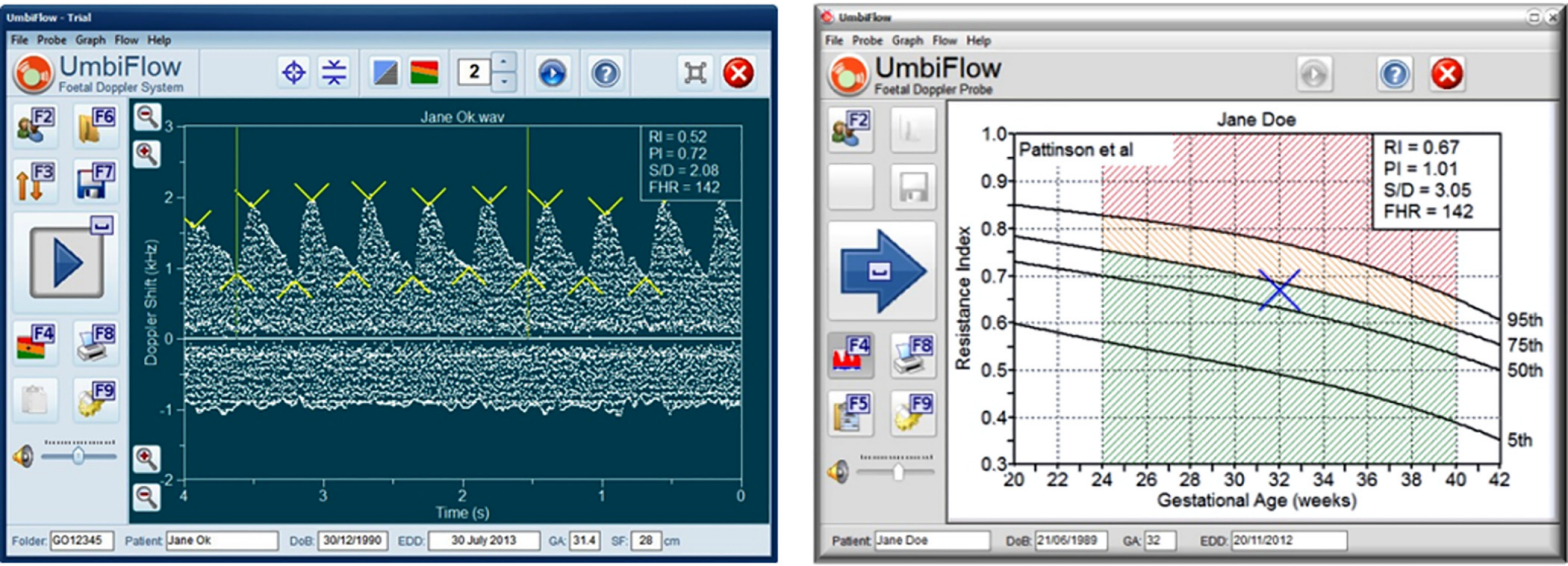
Results from an UmbiFlow™ Doppler measurement plotted against the gestation.

UmbiFlow™ has a simple user interface with a real-time visual representation of the flow in the umbilical artery and vein, and users are trained to recognise the correct pattern (Fig 1). In addition there is audio generation and users can learn to recognise the characteristic sound of the blood flowing through the umbilical artery. Once the umbilical artery is identified and the Doppler testing done, the software automatically generates a graph with the result plotted against the gestation (Fig 2). Three indices of systolic (S) to diastolic (D) flow are calculated in UmbiFlow™, namely the Resistance Index: RI = (S-D)/S; the Pulsatility Index (PI): PI = (S-D)/mean and the S/D Ratio (S/D). The reference values for the indices are those published by Pattinson et al [21] and validated for the Western Cape population. As far as the authors are aware, there is not currently a comparable device available.

In the Pattinson study, done with pulsed wave ultrasound machines, the RI showed the least inter-observer variation and was selected as the index of choice; it has been used in this population since then. The accuracy of the UmbiFlow™ system in measuring the RI in the umbilical artery has already been proven to be comparable to the commercial standard [22]. In subsequent studies by the same authors, the UA Doppler RI of ≥75^th^-95^th^ percentile showed a higher risk of perinatal mortality and the recommendation is that these women need more careful antenatal surveillance [23]. The purpose of the current study was to test the UmbiFlow™ system in a primary setting without immediate access to expensive pulsed wave Doppler or sophisticated ultrasonography.

The main aim of this pilot study was to determine whether it is feasible to use the UmbiFlow™at a primary care clinic to measure umbilical artery Doppler in patients with a suspected decreased SF growth; and whether this could safely lead to a decreased number of patients requiring referral to a more specialised level of care. Per definition, about 10% of low risk women will be referred to a Doppler (Fetal Evaluation) clinic with suspicion of fetal growth <10^th^ centile, as measured clinically (SF). Of these, yearly clinic statistics at Tygerberg shows that up to 80% could be referred back to their community clinic with normal UA Doppler. For lack of better terminology, these women will be called ‘false referrals’ in this study (as there is no placental insufficiency present). If they could receive their Doppler at primary care, it could decrease the workload at the tertiary hospital.

**Fig 3:**
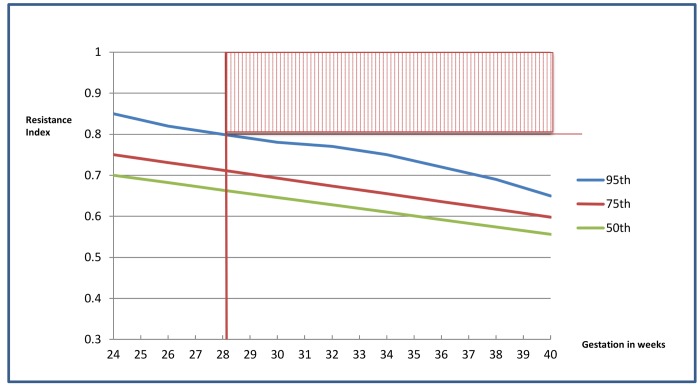
A schematic representation, showing that at any gestation ≥28 weeks, an umbilical artery resistance of >0.8 would be abnormal (≥95th centile) (shaded area).

In this low socio-economic setting, many patients book late with an uncertain gestation and thus serial SF measurement may be unreliable. A secondary aim of the study was to evaluate the feasibility of Doppler as a tool for detecting concealed intra-uterine growth restriction in late bookers by using a single screening cut-off value (RI of ≥0.8) that will be abnormal (≥95^th^ centile) for any gestation >28 weeks (see Fig 3).

## Materials and Methods

Pregnant women that attended the antenatal clinic between 1 August 2013 and 31 March 2014 were entered into the study, either as late new bookers (1^st^ visit UmbiFlow™ Doppler) or follow-up patients with suspected IUGR (decreased SF growth).

All patients with a decreased SF measurement underwent testing on site; performed by the midwife directly after the clinical examination. Midwifes were trained for 2 days in the use of UmbiFlow™ Doppler on-site before the start of the study. After that, a study nurse supported the midwives as an on-site consultant. Women with normal UmbiFlow™ Doppler for their respective gestation (defined as a RI <75^th^ centile according to existing protocols [24]); were re-assured and followed up as per routine. Women with UmbiFlow™ Doppler RI values ≥75^th^ but <95^th^ percentile for their specific gestation were referred to the doctor’s clinic at a district hospital, on the next working day, for confirmation of the value. The provincial ultrasound policy states that RI values ≥75^th^ but <95^th^ need only to be repeated two weeks later, but for safety reasons the study protocol requested that these values be confirmed at the next level of care. Women with clearly abnormal (≥95^th^ centile) values were directly referred to the tertiary hospital (Tygerberg) for assessment on the same day.

Measurements of the SF growth is taken with a tape measure and plotted on a customised growth chart at the best estimate for gestational age [25]. All clients that book before 24 weeks were referred for routine ultrasound to confirm gestational age, according to current provincial policy. This is a district level ultrasound examination, not a fetal anomaly scan, and focus on determining accurate gestation, the placental site and amniotic fluid volume. Full fetal anomaly scans are only offered at the tertiary centre for women with specific indications e.g. advanced maternal age or previous fetal anomaly.

All women who booked late (after an estimated 28 weeks of gestation) were assigned a gestation as well as could be calculated using routine means (dates, clinical examination and height of fundus). A single UmbiFlow™ Doppler RI measurement was done on them and they were referred if the value was ≥0.8.

The setting was Kraaifontein Midwife Obstetric Unit (MOU), situated in Cape Town in the Western Cape Province of South Africa and serving a large, low socio-economic community. It is situated 12km from Karl Bremer doctor’s clinic and district hospital, and 16km from its tertiary specialist referral hospital (Tygerberg Hospital).

The data was entered onto an anonymised Excel spreadsheet. Data from the actual UmbiFlow™ Doppler procedure (e.g. number of attempts, length of time taken to do the test) were remotely uploaded in real time via a 3G card to a cloud server, where the researchers could access the data. Data was analysed using standard software and expressed as means, standard deviations, medians and ranges. A pilot study over 6 months (of 137 consecutive patients) was conducted to be able to resource funding for a large field trial.

Approval for the prospective pilot study was obtained from the Stellenbosch University Health Research Ethics Committee (S12/10/263) as well as from the Western Cape Government. All patients consented to participation by signing a written consent form and they received an information leaflet. Permission to publish was obtained from the regional director of the Tygerberg substructure as well as from the chief executive officers of Karl Bremer and Tygerberg Hospitals.

## Results

**Fig 4:**
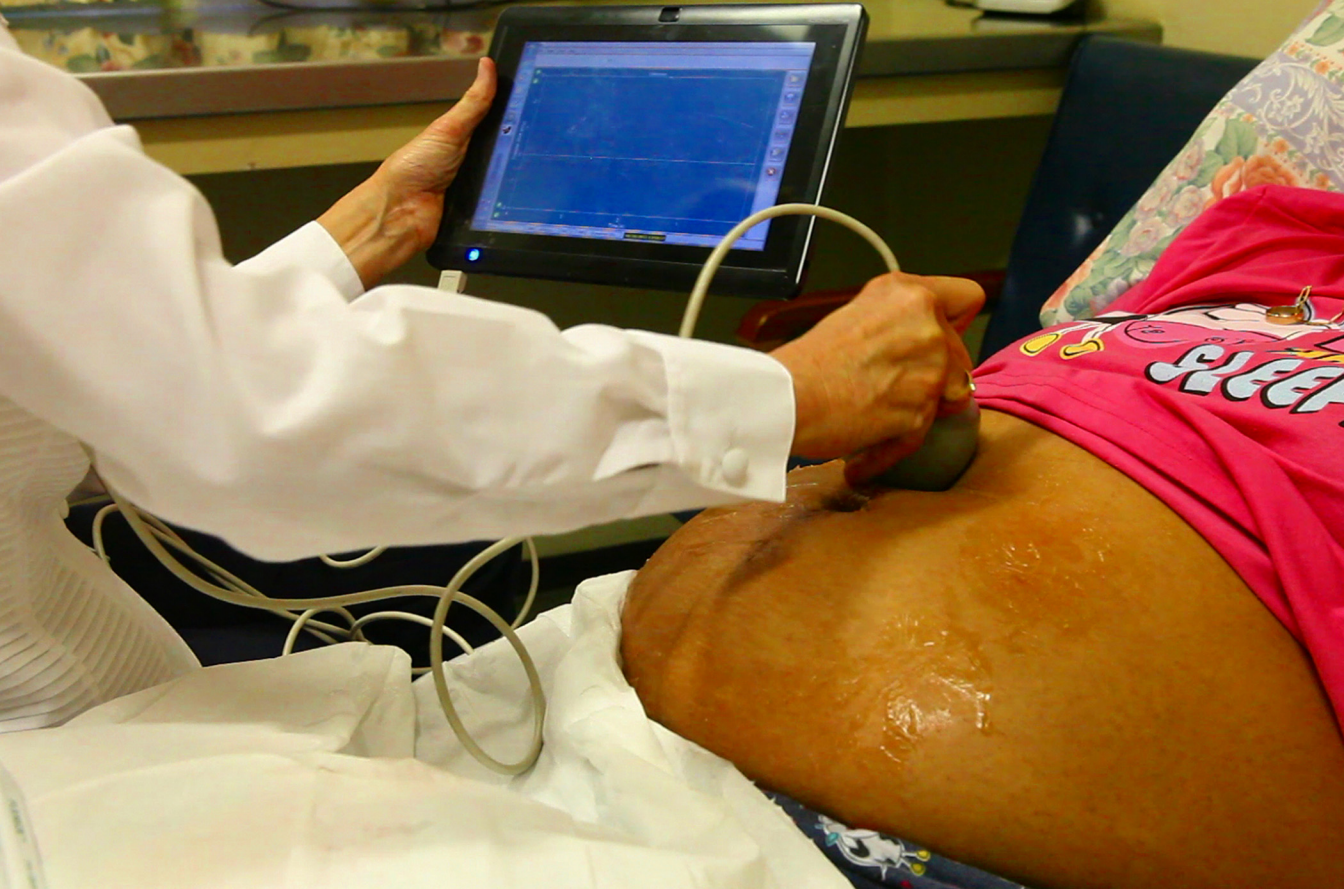
The UmbiFlow™ setup in a clinic.

UmbiFlow™ Doppler RI could be measured effectively by nurses who had not used this technique or software before, after training of only two days. A picture of the procedure is shown in Fig 4. There were 137 women included in the study, of which 129 folders were available for analysis; the other eight were lost to follow up. Thirty-one women entered the study due to poor SF growth and 106 were late bookers who received a single UmbiFlow™ Doppler test. The demographic characteristics are given in Table 1.

**Table 1:** Demographic characteristics of the study population.

	Poor SF Growth (n = 31)	Late Booker (n = 106)
Age in years (range)	25 (16–38)	24.9 (16–44)
Gravidity (minimum and maximum)	2 (1–5)	2 (1–6)
Parity (minimum and maximum)	1 (0–4)	1 (0–4)
Body Mass Index in kg/m^2^ (minimum and maximum)	23 (17–38)	26 (18–36)
Booking Haemoglobin (g/dl) (minimum and maximum)	11.5 (9–14)	10.6 (8.2–14)
Gestation at booking (minimum and maximum)	19 weeks 3 days (8–33 weeks)	30 weeks (28–36 weeks)

Of the 129 women where folders were available for analysis, only 22 were eligible for ultrasound referral (gestational age ≤24 weeks at booking) and 21 of them received on-site ultrasound gestational age determination as described earlier. There was a median of 8 days (range 0–69) difference between the estimated date of delivery as determined by clinical means (SF and/or dates) and the ultrasound confirmation.

Of the women with poor SF growth 12 (38.3%) had a completely normal UmbiFlow™ Doppler RI (<75^th^ centile) and could be re-assured without the need for referral. Nine of them still had a low SF at a follow up visit and was then referred for formal pulsed wave Doppler. Only six of them went for this evaluation and in all six cases the pulsed wave Doppler was also <75^th^ centile. Nine of the twelve had an uncomplicated post-natal outcome (at a mean gestation of 37 weeks) with a mean birth weight of 2553 gram (range 1890-3280g) and 5-minute Apgar of 9. Of the remaining three, one had a fetal death of a baby with anencephaly. This mother booked at 29 weeks and did not receive ultrasound screening. The second woman developed eclampsia at term and delivered a baby of 2250g, with good outcome. The third baby in this group had low birth weight baby (1890g), had two normal UmbiFlow™ Doppler RI measurements but the mother develop late onset pre-eclampsia. The case of anencephaly would not have been suspected on UmbiFlow™ Doppler, and the two late onset pre-eclampsia patients both had completely normal Doppler on the pulsed wave machine as well and were not referred for additional Doppler test (uterine artery and anomaly scan) as it is not currently provincial policy.

A further 11 women (35%) had UmbiFlow™ Doppler RI values ≥75^th^ but <95^th^ centile and was referred to the doctors clinic for confirmation. Four were referred back with normal pulsed wave Doppler values and six were retained at the district level due to abnormal pulsed wave Doppler RI (n = 3) or maternal anaemia (n = 4), including one with a breech presentation. One woman did not return for any follow up visit or pulsed wave Doppler test. She delivered her baby at home at 35 weeks (1665g, the baby was admitted for prematurity). The remaining women delivered (at a mean gestation of 39 weeks) with mean birth weight of 2800g (2260-3530g).

Six patients with decreased SF growth had clearly abnormal UmbiFlow™ Dopplers (RI ≥95^th^ centile) and were referred for further management. One had severe IUGR with subsequent fetal growth <1^st^ centile and the eventual birth weight 2230g. Three had fetal compromise necessitating Caesarean section (CS) with birth weights 1135g at 31 weeks, 1800g at 37 weeks and 2275g at term. The remaining two patients developed complications of hypertension and delivered a 2810g and 2010g baby respectively, both at 37 weeks.

**Fig 5:**
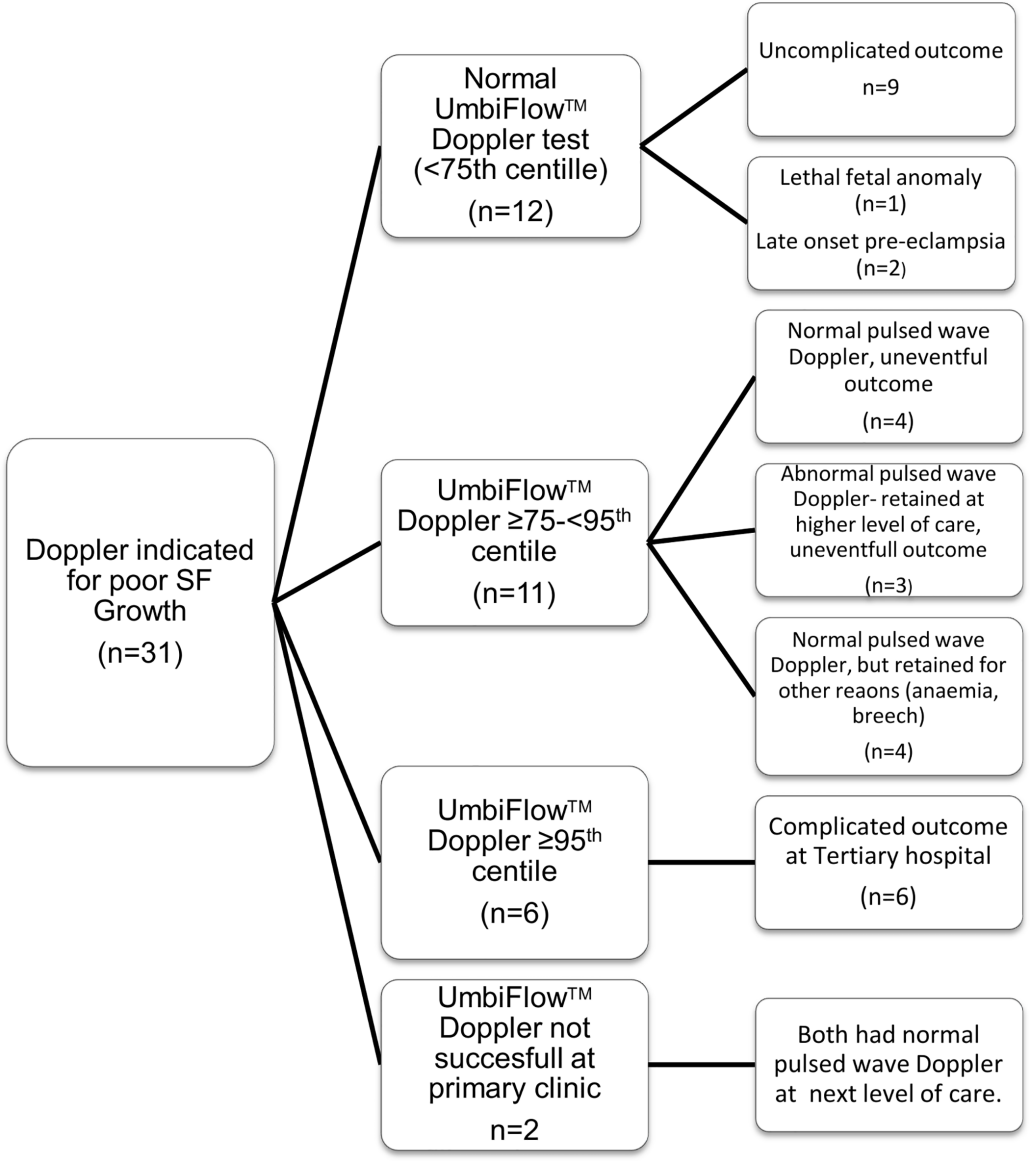
Algorithm of participating women in the poor SF group.

Only three women in this group admitted to smoking tobacco products and one was referred for cannabis misuse. The nurses were unable to get a satisfactory tracing in two women (6.5%) and they were referred to the district hospital service. One had a normal pulsed wave Doppler and was referred back and the other one was induced for hypertension on the day of the referral. An algorithm describing the outcome of participating women is shown in Fig 5.

Of the late bookers, 106 were enrolled for the study. These women were completely low risk and healthy as those with high risk criteria were referred directly after booking. Eight women (7.5%) had a random UmbiFlow™ Doppler RI value of ≥0.8 and were referred. Two had a much earlier gestational age after re-calculation with ultrasound (and therefor acceptable RI) and were referred back; they had an uneventful pregnancy outcome. Of the remaining six, two had a normal pulsed wave Doppler at the referral hospital but both had an eventful outcome. One developed late onset pre-eclampsia (birth weight 2620g) and the other ante-partum haemorrhage (birth weight 3190g). Three had pulsed wave Doppler RI ≥75^th^ -<95^th^ centile and were retained at district hospital level but were otherwise uncomplicated. Their mean birth weight was 2703g. The remaining patient had a RI ≥95^th^ centile and developed antepartum haemorrhage. Her baby weighed 2410g. The birthweight in the group with a RI ≥8 was lower (mean 2742g) than the group <0.8 (mean 3072g) but the difference was not significant.

There were ten women where the attending nurses could not obtain a good Doppler pattern to measure. The protocol did not include upward referral for these cases, but four were referred for Doppler during their subsequent antenatal care. Two had a RI of ≥75^th^ -<95^th^ centile and one a normal initial Doppler but later developed IUGR and abnormal Doppler. The fourth was seen on two occasions where Doppler was unsuccessful, at the last visit she complained of decreased fetal movements and on arrival at the referral hospital there was an intra-uterine death (IUD). A macerated stillbirth (birthweight 1640g) was delivered and the placental histology showed placental insufficiency.

**Fig 6:**
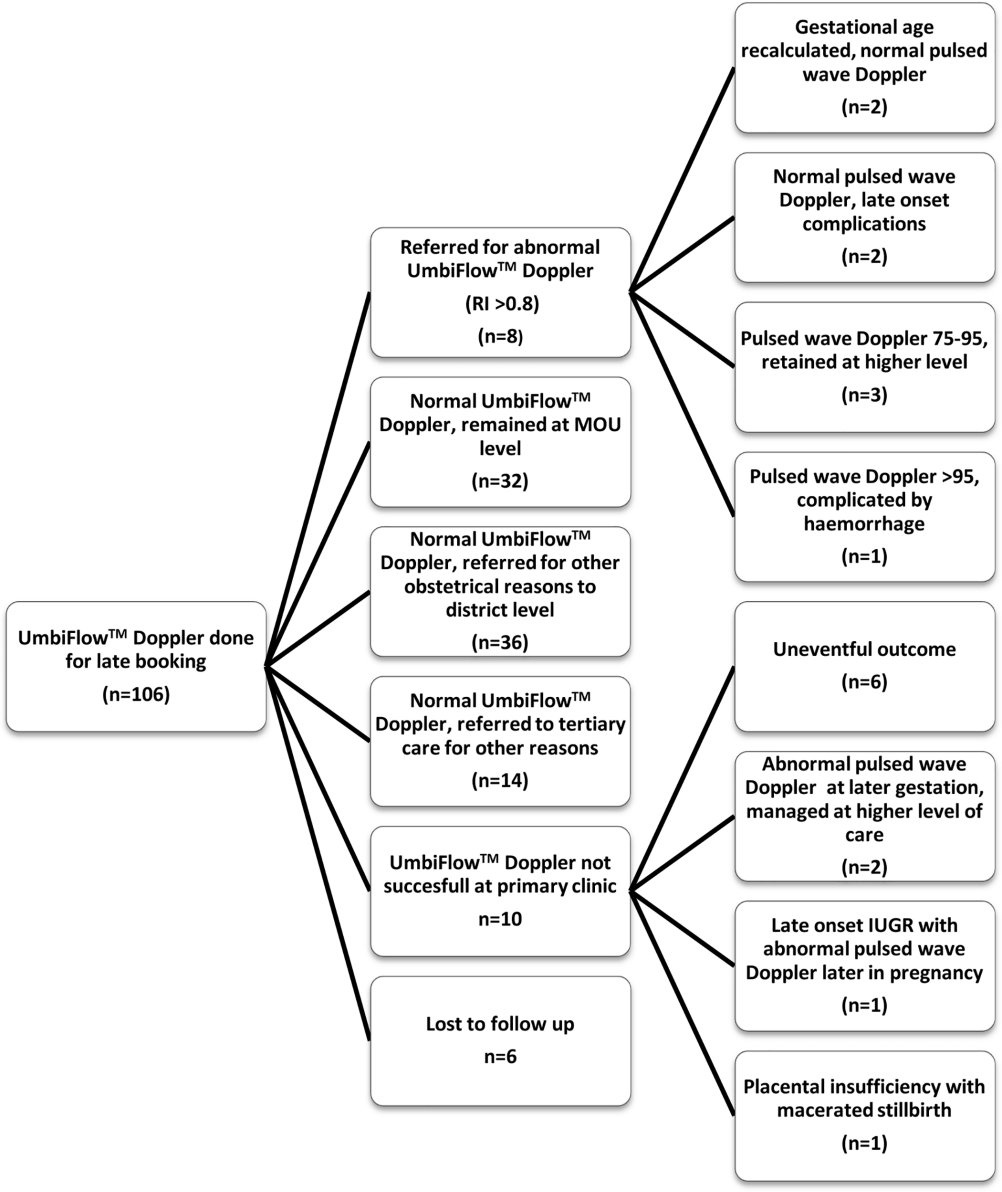
Algorithm of participating women in the late bookers group.

Of the remaining (n = 88) with Doppler RI <0.8; thirty-two (30.2%) remained at midwife level of care and delivered at the MOU. A further thirty-six (33.9%) continued with care at the MOU but delivered at a district hospital, for complications not necessarily associated with impaired SF growth- induction of labour for post-dates, CS for fetal distress during labour, poor progress during labour, trial of labour after previous CS, late onset pre-eclampsia, preterm labour and maternal anaemia. Fourteen of the 88 were referred to a tertiary institution later during their pregnancies. The reasons for referral were preterm labour or late onset pre-eclampsia. The delivery pattern for all women in Cape Town is 32.5% at primary level, district hospital referral in 55.6%, referral to specialist (regional) care 15.3% and delivery at tertiary centres 7.8% (provincial delivery data), so the referrals was not unexpected. The mean gestation at the time of delivery was 37 weeks (range 32–42) and the mean birth weight in this group was 2777 g (range 2060-3710g). All the babies were delivered alive and there was no case of late-onset IUGR amongst them. In this group there were six women that were lost to follow up. An algorithm describing the outcome of participating women is shown in Fig 6.

Out of the whole group, 49 women were referred to the district hospital for Doppler repeat tests. Of these, seven (14.3%) did not attend their appointment. Because of its mobile-connectivity, all results (including monitoring of the entire field trial, e.g. the exact time the nurse spent on the procedure) could be assessed and evaluated.

## Discussion

This is the first field trial of a locally developed, low-cost mobile umbilical artery Doppler device in a primary care setting where the attending nurse practitioner performed the tests as part of clinical practice. A successful test could be performed in more than 90% of women. UmbiFlow™ Doppler correctly identified all the women in the poor SF growth group who had significant pathology. There was a high incidence of pathology when the nurses could not get a satisfactory tracing in late bookers (including one IUD). An important finding of this trial is that those women should be referred for ultrasound-directed Doppler as soon as possible.

Referral to specialised services could be avoided in twelve women with perceived decreased fundal growth without any complications. If all of the Doppler tests described in the poor SF group was done in a hospital setting, 22 women (the 12 identified by UmbiFlow™ Doppler plus the 2 where UmbiFlow™ Doppler could not get an initial tracing plus the 8 who had an initial raised UmbiFlow™ Doppler but subsequent normal pulsed wave Doppler at the next level of care, see Fig 5) would have had a normal Doppler and be referred back (if there was no other reason for retention at a higher level of care). Thus UmbiFlow™ Doppler done at primary care could prevent 12/22 or 55% of unnecessary referrals (false-referrals).

In a previous study in this population group, about 5% of low risk women were referred for poor SF growth [12]. In Cape Town, where 62 285 women booked at antenatal clinics in 2014, if 5% (n = 3114) was referred for suspected poor fetal growth to hospital clinics, UmbiFlow™ Doppler done at primary care could have reduce the number of false-referrals with 1712 women (55%).

If all women with a UA Doppler <95^th^ centile (n = 23) were retained at primary care, 74% of the initial referrals could be avoided. It remains important to be vigilant for late onset hypertensive disease following a normal umbilical artery Doppler. Only 85% of women referred for Doppler to the next level of care arrived for their appointments. The point-of-care testing for a potential poor outcome ensured 100% attendance at the local clinic. There was clear pathology in the women with poor fundal growth and UA Doppler ≥95^th^ centile and these women could be referred timeously for appropriate management.

The incidence of late-onset IUGR is low and large numbers of women will be needed to show if a random UA Doppler test is of value in late bookers. In this pilot study, only 7.5% of women (n = 8) had a test that warranted onward referral. The referred women had a non-significant lower birth weight but whether this is related to premature iatrogenic intervention or a real growth concern or pathology is uncertain.

In the intervening years since UmbiFlow™ Doppler was developed, ultrasound technology has also advanced and less expensive machines equipped with pulsed wave Doppler are available on the market. In an ideal scenario, real time ultrasound with pulsed wave Doppler will identify the correct place where the Doppler measurement should be done (at the insertion of the umbilical cord into the placenta) and have the added advantage of evaluating the baby for gross abnormalities, placental insertion, amniotic fluid and basic gestation. UmbiFlow™ Doppler is less sophisticated and generates a waveform only. Users are trained to detect the correct soundwave by ear as well as visualise a correct wave pattern on the screen. In a developing country, ultrasonographers are in scarce supply and the possibility to train midwifes (another scarce resource) to do basic ultrasound, though promising, is not yet feasible on a wider scale [26]. Until a full scale-up to ultrasound in rural clinics are available (highly unlikely in a developing country) UmbiFlow™ Doppler fills this middle ground as it is cheap, the skill can be imparted to a midwife within 2 days and the small device can be incorporated into the clinic without adding much more time to the consultation. A possible risk of not referring women with poor fundal growth and normal UmbiFlow™ Doppler is that other incidental pathology such as fetal anomalies, late onset intra-uterine growth restriction or low lying placenta could be missed. These issues should rather be addressed with early booking and routine referral for detailed ultrasound than to rely on chance pick-up during later referrals. The fetal evaluation clinic at Tygerberg is also midwife managed and women referred for umbilical artery Doppler will not receive ultrasound evaluation unless it is indicated according to the provincial ultrasound policy. Shifting this midwife service to the community will not therefore impart more danger for the non-referred women.

### Conclusion

The feasibility of introducing a low cost mobile umbilical artery Doppler device used by nurse practitioners in a primary care antenatal clinic as a screening test for chronic placental insufficiency when IUGR is suspected and with late bookers have been proven. The results of the pilot study will be used to validate the relevance of this strategy in a larger population; appropriately powered to determine possible significant advantages of Doppler used by nurse practitioners as a point of care test.
